# Radiation Recall Pneumonitis Induced by Sintilimab: A Case Report and Literature Review

**DOI:** 10.3389/fimmu.2022.823767

**Published:** 2022-02-23

**Authors:** Min Wang, Shuhui Xu, Hui Zhu

**Affiliations:** ^1^ Department of Radiation Oncology, Shandong Cancer Hospital and Institute, Shandong First Medical University and Shandong Academy of Medical Sciences, Jinan, China; ^2^ Department of Radiation Oncology, Shandong Cancer Hospital and Institute affiliated to Shandong University, Jinan, China

**Keywords:** radiation recall pneumonitis, radiation pneumonitis, NSCLC, immune checkpoint inhibitors, radiotherapy

## Abstract

Radiation recall pneumonitis (RRP) is described as an unpredictable acute inflammatory reaction within the previously irradiated lung site during the administration of systematic therapy after radiotherapy. Here, we reported a case of a 54-year-old woman with non-small lung cancer (NSCLC), who had pneumonitis at 3 and 10 months after radiotherapy regarded as radiation pneumonitis (RP) and RRP induced by anti-PD-1 sintilimab, respectively. This unique patient with double pneumonitis (RP and RRP) has drawn attention to the identification of immune or radiation pneumonitis, its potential mechanism, and further treatment strategy after the emergence of RRP.

## Introduction

Radiation pneumonitis (RP) is an acute inflammation that occurs within 6 months, most often within 12 weeks, after the end of radiation therapy (RT) (1, 2). Radiation recall pneumonitis (RRP) is an unpredictable acute inflammatory reaction within the previously irradiated lung area during the course of systematic therapy (3, 4). RRP is mainly associated with chemotherapeutic drugs and EGFR-tyrosine kinase inhibitors (TKIs) (4–7), whereas RRP induced by immune checkpoint inhibitors (ICIs) has been rarely reported.

The combinations of RT and ICIs have shown significant benefits in cancers. For example, durvalumab has been approved as consolidation therapy after chemoradiotherapy in patients with unresectable stage III non-small lung cancer (NSCLC). Moreover, a pooled analysis of the results of the PEMBRO-RT and MDACC trials reported that the combination of pembrolizumab and RT enhanced both overall survival (OS; *p* = 0.0004) and progression-free survival (PFS; *p* = 0.045) in patients with advanced NSCLC. However, the incidence of immune-related pneumonitis was found to range from 0% to 5.8%, indicating that the efficacy of RT and immunotherapy is limited by lung toxicity (8).

Administration of ICIs after RT may lead to the development of RRP through toxic overloading or triggering memory responses. A subgroup analysis from the PACIFIC trial (9) found that any grade pneumonitis was more frequent in patients who were treated than those not treated with durvalumab after RT (33.9% vs. 24.8%). A secondary analysis of the KEYNOTE-001 phase I trial showed that a history of RT before pembrolizumab was predictive of longer PFS but was associated with a higher incidence of pneumonitis (13% vs. 1%, *p* = 0.046) (10). Similarly, the incidence of pneumonitis in the PEMBRO-RT trial was higher in patients who received pembrolizumab after RT than those who received pembrolizumab alone (26% vs. 8%) (11).

## Case Report

**Figure 1:**
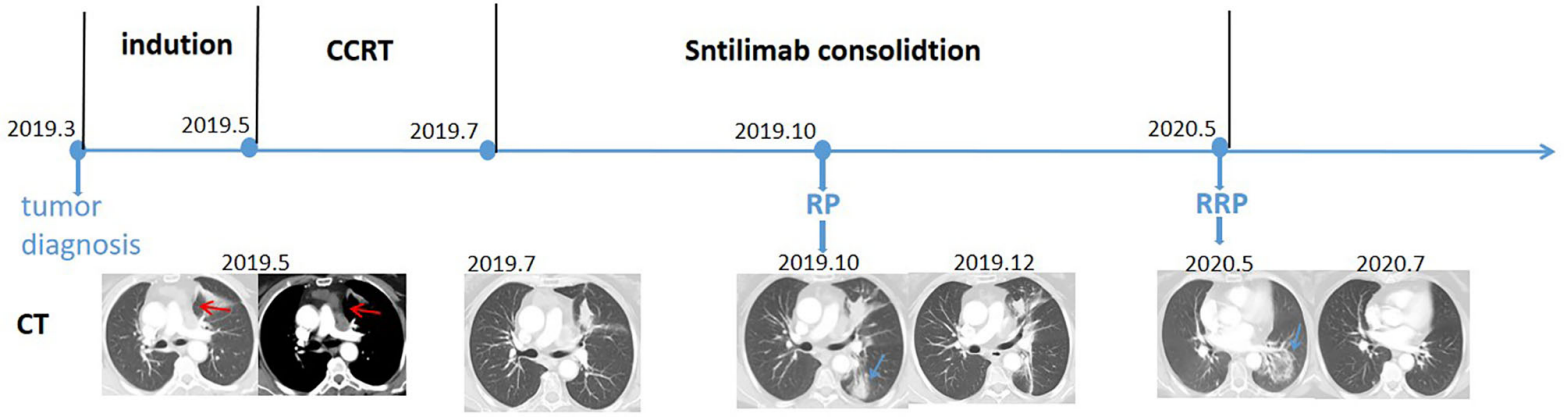
Timeline of the major treatment process and CT evaluation of the patient since diagnosis. CT, computed tomography; CCRT, concurrent chemoradiotherapy; RP, radiation pneumonitis; RRP, radiation recall pneumonitis. Red arrow: tumor site; blue arrow: pneumonitis site.

**Figure 2:**
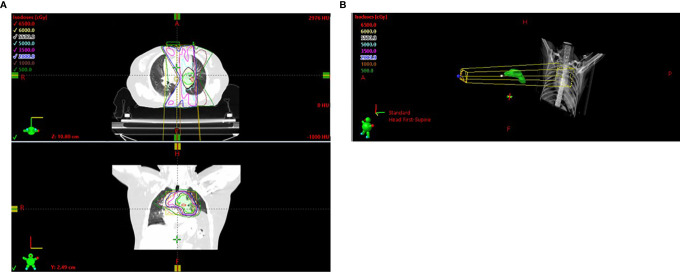
**(A)** Radiation field. **(B)** DRR in the AP. DRR, digitally reconstructed radiograph; AP, anterior–posterior.

In March 2019, a 54-year-old woman with an Eastern Cooperative Oncology Group (ECOG) score of 1 presented with hoarseness and cough without an obvious cause. An enhanced chest CT scan revealed a nodule in the left upper lobe and mediastinal lymph node metastases in the pulmonary artery. Bronchoscopy revealed a pathological diagnosis of lung adenocarcinoma. Brain MRI scan and whole-body bone scan did not reveal other sites of metastatic disease. Preliminary staging of the tumor determined it to be cT2N2M0 IIIA. According to the multidisciplinary team (MDT), patients with pulmonary artery invasion cannot be treated surgically, with concurrent chemoradiotherapy (CCRT) being the standard treatment. Therefore, the patient was enrolled in a randomized, controlled phase III clinical trial evaluating the use of sintilimab as consolidation therapy in patients with unresectable, locally advanced NSCLC (stage III) without disease progression after radical CCRT. The patient received two cycles of induction chemotherapy which ended in May 2019, followed by CCRT which ended in July 2019. The gross tumor volume (GTV) included the lung lesion and metastatic lymph nodes, with the GTV and 0.8-cm margins yielding the clinical tumor volume (CTV) and the CTV with additional 0.5-cm margins yielding the planning tumor volume (PTV). CCRT consisted of 30 fractions of 2.0 Gy each, for a total of 60.0 Gy (**Figure 2A**). The digitally reconstructed radiograph (DRR) in the anterior–posterior (AP) is shown in **Figure 2B**. The mean lung dose (MLD) was 13.5 Gy, with 17% of the lung receiving a dose of 20 Gy (V20) and 53% of the lung receiving a dose of 5 Gy (V5). Chest CT after CCRT showed a partial response (PR) (30.3% lesion reduction), with the patient subsequently receiving consolidation sintilimab every 3 weeks. The major treatment process and CT evaluation of the patient since diagnosis is shown in **Figure 1**.

Three months after RT, during the third cycle of sintilimab treatment, the patient experienced pneumonitis with a slight cough, with imaging showing consolidation in the left lower lobe (**Figure 1**). According to CTCAE 4.0, this pneumonitis was diagnosed as a mild form of grade II RP. Taking into consideration the patient’s request, she was treated with traditional Chinese medicine (a simplified formula of Baihegujin decoction including raw ground Radix Scrophulariae, *Paeonia lactiflora*, and Sichuan shell) to relieve her cough and continued to be treated with sintilimab. CT reexamination showed improvement 2 months after the first episode of pneumonitis, and it continued to show PR (32% reduction of tumor lesion). Because the time interval between the end of RT was short (<6 months) and the patient continued to respond to sintilimab, the first episode of pneumonitis was defined as RP.

At 10 months, during the 10th cycle of sintilimab therapy after RT, the patient experienced severe dyspnea and cough. A chest CT scan showed a new ground-glass opacity in the left lower lobe, which localized within the previously irradiated area (**Figure 2**). No evidence of significant infection was found in the blood and sputum cultures. Based on CT imaging and long-time interval, the second episode of pneumonitis was not likely to be conventional RP or immune-related pneumonitis; rather, it was regarded as RRP induced by sintilimab. Sintilimab treatment was discontinued in this patient, and she was rather started on 120 mg q12h prednisone, which was gradually tapered over more than 4 weeks. Her symptoms were gradually relieved and CT reexamination showed obvious improvement after 2 months without tumor progression (**Figure 1**).

## Discussion

The present study describes a patient who experienced RRP induced by sintilimab, a whole humanized IgG4 monoclonal antibody that blocks the interaction of PD-1 with its ligands PD-L1 and PD-L2.

To date, there is no general consensus regarding the diagnosis of RRP. In general, RRP is regarded as pneumonitis not due to other causes, including RP, antitumor-induced pneumonitis, pulmonary infection, and progressive tumor. Although the initial clinical manifestations of RRP included fever, cough, and dyspnea, these clinical symptoms can occur in all types of pneumonitis and may serve as warning signs rather than for differential diagnosis. Imaging changes limited to previously irradiated areas resulting in ground-glass opacity without tumor progression are also indicative of RRP, whereas immune-associated pneumonitis is not limited to high-dose areas (12). Moreover, RRP usually occurs in patients taking antitumor agents after RT. Another difference between RP and RRP is that RP usually occurs within 6 months of RT, whereas RRP occurs at later times (13). Finally, blood culture and blood sample examination can distinguish RRP from pulmonary infections.

Treatments for RRP include cessation of antitumor agents and application of corticosteroids and corresponding supportive care (3). The efficacy of rechallenge with the same antitumor agents is unclear. The RRP recurrence rate after rechallenge with the same ICI was reported to be 28.8% (14). Interestingly, pneumonitis was associated with a higher recurrence rate (odds ratio, 2.26; 95% confidence interval, 1.18–4.32; *p* = 0.01). Hence, rechallenge after RRP requires careful evaluation by the MDT. Because the present patient was at high risk for recurrent sintilimab-induced pneumonitis, this agent was discontinued. Additional studies are needed to determine the standard treatment for RRP.

The apparent benefits of ICI after RT have led to increases in the number of patients with a variety of cancers receiving ICIs. Clinicians should carefully evaluate cancer patients at risk of ICI-induced RRP.

## Data Availability Statement

The original contributions presented in the study are included in the article/supplementary material. Further inquiries can be directed to the corresponding author.

## Ethics Statement

Written informed consent was obtained from the individual(s) for the publication of any potentially identifiable images or data included in this article.

## Author Contributions

HZ: conceptualization and methodology. MW: writing—original draft preparation. SHX: data curation, visualization, and investigation. All authors read and approved the final manuscript.

## Funding

This work was supported by CSCO-Pilot Cancer Research Fund (grant number: Y-2019AZZD-0352) and Key Research and Development Program of Shandong Province (grant number: 2018GSF118067).

## Conflict of Interest

The authors declare that the research was conducted in the absence of any commercial or financial relationships that could be construed as a potential conflict of interest.

## Publisher’s Note

All claims expressed in this article are solely those of the authors and do not necessarily represent those of their affiliated organizations, or those of the publisher, the editors and the reviewers. Any product that may be evaluated in this article, or claim that may be made by its manufacturer, is not guaranteed or endorsed by the publisher.
